# Pevonedistat, a Nedd8-activating enzyme inhibitor, sensitizes neoplastic B-cells to death receptor-mediated apoptosis

**DOI:** 10.18632/oncotarget.15050

**Published:** 2017-02-03

**Authors:** Cody Paiva, J. Claire Godbersen, Taylor Rowland, Olga V. Danilova, Christopher Danes, Allison Berger, Alexey V. Danilov

**Affiliations:** ^1^ Knight Cancer Institute, Oregon Health and Science University, Portland, OR, USA; ^2^ Geisel School of Medicine at Dartmouth, Department of Medicine, Hanover, NH, USA; ^3^ VA Portland Healthcare System, Department of Pathology and Laboratory Medicine, Portland, OR, USA; ^4^ Millennium Pharmaceuticals, Inc., a wholly owned subsidiary of Takeda Pharmaceutical Company Ltd., Cambridge, MA, USA

**Keywords:** lymphoma, neddylation, CLL, TRAIL

## Abstract

While death receptor ligands (Fas and TRAIL) kill chemoresistant tumor cell lines, related therapies have limited clinical efficacy as single agents. Death receptor signaling is modulated by nuclear factor-κB (NFκB), a family of transcription factors which are constitutively active in B-cell malignancies. We and others have shown that pevonedistat, an investigational inhibitor of the NEDD8-activating enzyme, abrogates NFκB activity in B-cell neoplasia. Here we demonstrate that diffuse large B-cell lymphoma, particularly activated B-cell type, and primary chronic lymphocytic leukemia cells are re-sensitized to extrinsic apoptosis by pevonedistat. Pevonedistat enhanced caspase-8 processing following death receptor ligation, and downmodulated cFLIP, a NFκB-regulated protease-deficient caspase homolog. However, treatment with pevonedistat did not modulate death-inducing signaling complex in neoplastic B-cells, suggesting that they were sensitized to death ligands through the mitochondrial pathway. Our data provide rationale for further development of pharmacologic agents including pevonedistat in strategies which enhance death receptor signaling in lymphoid malignancies.

## INTRODUCTION

Apoptotic pathways are dependent on activation of initiator and executioner caspases (caspase-2/8/9/10 and 3/6/7, respectively). Caspase activation can be induced at the plasma membrane (extrinsic apoptosis) or at the mitochondria (intrinsic apoptosis). Engagement of death receptors belonging to the tumor-necrosis factor (TNF) receptor gene superfamily, e.g. TRAIL-R1/2 (TNF-related apoptosis-inducing ligand receptors; DR4/5) and Fas (CD95) by their respective cognate ‘death ligands’ TRAIL (Apo2L) and FasL (Apo1L), kills multiple tumor cell lines independent of their chemosensitivity or *TP53* mutational status [1–5]. While Fas is highly toxic against hepatocytes and can induce fulminant liver injuries [6], TRAIL lacks toxicity in animal models, thus holding promise in oncology therapeutics. Whereas a number of preparations of TRAIL and its derivatives were safe in clinical trials, single agent efficacy data is disappointing, necessitating the development of novel combination approaches [4].

Among the factors which contribute to resistance to death ligands, the nuclear factor-κB (NFκB)-driven upregulation of the anti-apoptotic genes in response to death receptor ligation was shown to result in a diminished cellular susceptibility to extrinsic apoptosis across several tumor types [7–9]. The NFκB transcription factors modulate cell survival during stress and immune response [10]. Their anti-apoptotic function is fulfilled in part via regulation of the inhibitor of apoptosis (IAP) and Bcl-2 family members. Recent reports added controversy to the role of NFκB in death receptor signaling, where individual NFκB subunits were shown to play conflicting roles [11]. For example, the predominantly pro-survival activity of the RelA (p65) may be counterbalanced by pro-apoptotic effect of c-Rel.

NFκB pathway deregulation contributes to oncogenesis in B-cell malignancies and is detected in both aggressive (diffuse large B-cell lymphoma [DLBCL]) and indolent (chronic lymphocytic leukemia/small lymphocytic lymphoma [CLL]) non-Hodgkin lymphoma (NHL) subtypes [12, 13]. Gene expression profiling categorizes DLBCL based on cell-of-origin, where NFκB activation is the key feature of the less curable activated B-cell-like (ABC)-DLBCL [14]. However NFκB aberrations are also found in germinal center-like (GC)-DLBCL [12]. We and others have established that pevonedistat (MLN4924, TAK-924), an investigational inhibitor of the NEDD8-activating enzyme (NAE), successfully abrogates NFκB pathway activity in B-cell malignancies [15–17]. Interaction between NAE and NEDD8, a ubiquitin-like modifier, ultimately leads to activation of Cullin-RING ligases (CRL), followed by ubiquitination and degradation of their substrate proteins. Pevonedistat forms a covalent adduct with NEDD8, thereby disrupting this interaction, and leading to extended half-life of CRL substrates, including inhibitor of NFκB (IκB) [15, 18]. Recent clinical data shows that pevonedistat has a favorable adverse event profile in patients with hematologic malignancies [19, 20].

Given the pathogenic role of NFκB in lymphoma, and its role in resistance to death ligands, we studied whether NAE inhibition sensitizes neoplastic B-cells to extrinsic apoptosis.

## RESULTS

### NAE inhibition sensitizes neoplastic B-cells to extrinsic apoptosis

**Figure 1:**
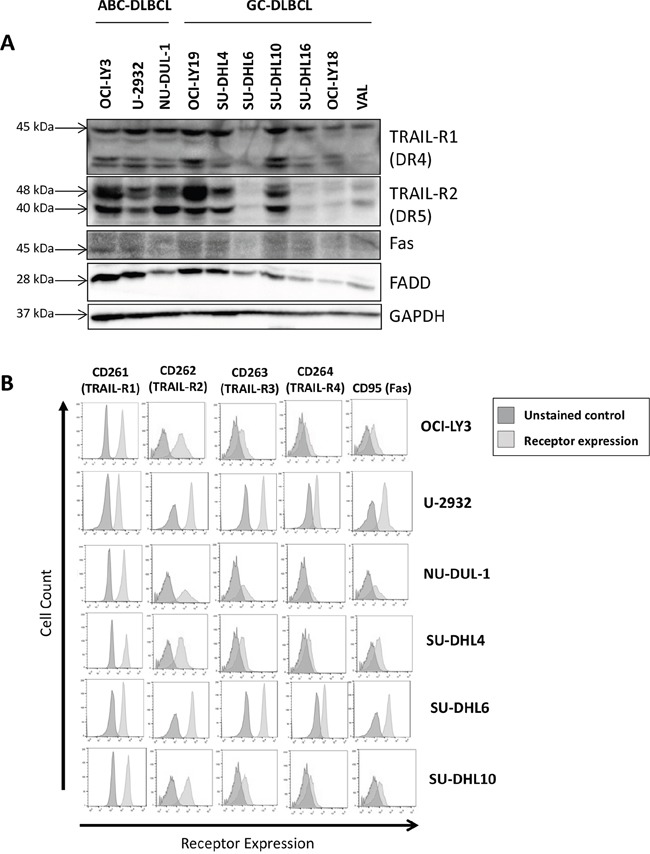
Death receptor expression in DLBCL cell lines was determined in whole-cell protein lysates by immunoblotting **A.** and by flow cytometry **B**.

We studied expression of TRAIL-R and Fas (CD95) in a panel of DLBCL cell lines. TRAIL-R1 (DR4) was expressed in all tested DLBCL cell lines, while TRAIL-R2 (DR5) was highly expressed in ABC-DLBCL and in 3/7 tested GC-DLBCL cell lines (Figure 1). By contrast, Fas was expressed at low levels, while Fas-associated death domain (FADD) adaptor protein was detectable in all DLBCL cell lines (Figure 1A). Cell surface expression of TRAIL-R1/2 and Fas was confirmed by flow cytometry (Figure 1B). “Decoy” receptors TRAIL-R3/4, which are unable to transmit apoptotic signals and thus may foster resistance to TRAIL-mediated apoptosis [21], were expressed at low levels (Figure 1B).

**Figure 2:**
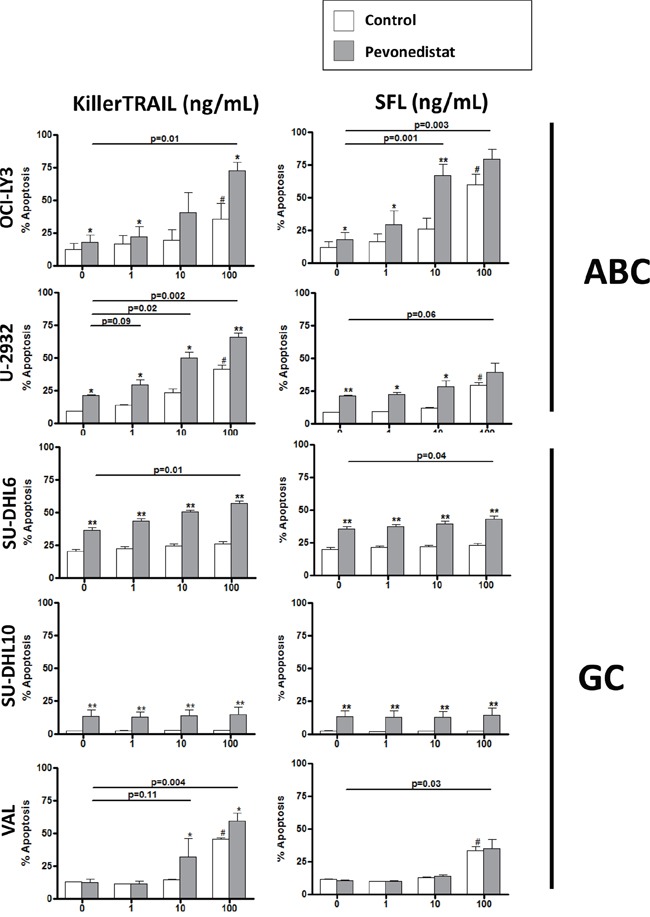
Pevonedistat sensitizes DLBCL cells to death receptor agonists Cell lines were incubated with the indicated concentrations of KillerTrail and SuperFasLigand (SFL) and 0.5 μM pevonedistat (or vehicle control) for 24 hours. Apoptosis was determined by Annexin V staining. Data are the mean ± SE of three independent experiments. ^#^ - p<0.05 compared to untreated control; * - p<0.05 and ** - p<0.01 when comparing death receptor agonist/pevonedistat combination with the agonist alone.

Despite this, DLBCL cells were resistant to both TRAIL and Fas ligand used in concentrations sufficient to induce killing of Jurkat cells (up to 10 ng/mL, data not shown and [22, 23]; Figure 2 and Supplementary Figure 1). Exposure to high concentration of ligands (100 ng/mL) led to minimal cell apoptosis (Figure 2).

Meanwhile, NAE inhibitor pevonedistat sensitized DLBCL cells to death receptor agonists. Pevonedistat alone used at a clinically achievable concentration of 0.5 μM [19], induced <20% apoptosis, in both ABC- and GC-DLBCL cells (Figure 2, gray bars on the left). However, ABC-DLBCL cells were sensitized to death receptor signaling (Figure 2 and Supplementary Figure 1). Of note, 68.5±5.2% OCI-LY3 cells underwent apoptosis following treatment with pevonedistat combination with 10 ng/mL Fas ligand, versus 25.2±4.7% with Fas ligand alone. Pan-caspase inhibition rendered partial protection from apoptosis (Supplementary Figure 2). By contrast, GC-DLBCL cells exhibited modest, if any, sensitization (Figure 2).

Thus, DLBCL cells were resistant to extrinsic apoptosis irrespective of the cell of origin, while pevonedistat sensitized DLBCL cells (predominantly ABC-like) to death receptor agonists.

### Targeting NAE enhances extrinsic apoptosis in CD40-stimulated primary CLL cells

**Figure 3:**
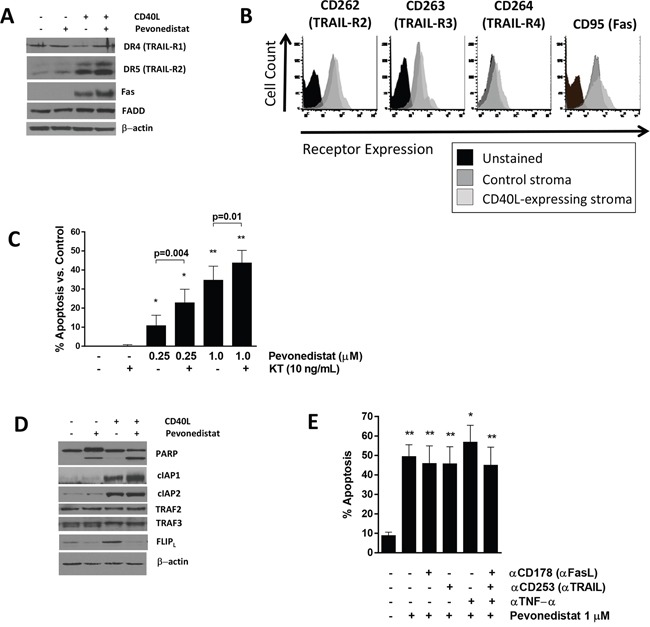
Pevonedistat sensitizes CLL cells to death receptor agonists **A** *and* **B.** CLL cells were co-cultured with CD40L-expressing or control stroma for 24 hours and analyzed by flow cytometry (B), followed by incubation with 1 μM pevonedistat or vehicle control for 24 hours. Cells were lysed and subjected to immunoblotting. A representative blot from 4 independent experiments is shown. **C.** CLL cells (N=6) were co-cultured with CD40L-expressing for 24 hours and subsequently incubated with the indicated drugs for 24 hours. Apoptosis was determined by Annexin V and 7-AAD staining within the CD19^+^ subset of cells. Data are mean±SE. Values were normalized to untreated control. **D.** CLL cells were treated as in (A). **E.** CLL cells (N=4) were co-cultured with CD40L-expressing for 24 hours and then treated with 1 μM pevonedistat, the indicated antibodies (0.5 μg), or the combination for 24 hours. Apoptosis was determined by Annexin V and 7-AAD staining within the CD19^+^ subset of cells. Data are mean±SE. * - p<0.05, ** - p<0.01 compared with untreated control.

We then studied whether our findings could be replicated in primary neoplastic B-cells. Our group has previously demonstrated that targeting NAE potently inhibits NFκB in primary CLL cells under the conditions mimicking the lymph node microenvironment [15, 16]. Here, as previously, CLL cells were cultured in the presence of CD40L-expressing stroma, leading to activation of the canonical and non-canonical NFκB pathways, and resistance to spontaneous and drug-induced apoptosis, as previously shown by us and others [15, 24]. Death receptors and their ligands, notably Fas, FasL and TRAIL, are regulated by NFκB [25–27]. While unstimulated CLL cells derived from peripheral blood had low death receptor expression, CD40L stimulation upregulated Fas and TRAIL-R2 (but not TRAIL-R1) as well as “decoy” receptors TRAIL-R3/4 in CLL cells (Figure 3A-3B). Despite this, treatment with TRAIL or FasL alone did not kill CLL cells (Figure 3C). Meanwhile, NAE inhibition did not affect death receptor expression (Figure 3A), but resulted in sensitization of the CD40-stimulated CLL cells to TRAIL (Figure 3C).

In agreement with our earlier findings [15], CD40L-stimulated CLL cells were sensitized to pevonedistat compared to cells co-cultured with stroma control (Figure 3D). Given that autologous cytotoxic T-lymphocytes, NK cells and monocytes present in co-cultures could secrete endogenous death receptor ligands, we inquired whether enhanced death receptor expression could explain this finding. However, neutralizing antibodies against TNFα, FasL (CD178) and TRAIL (CD263), alone or in combination, did not render protection from pevonedistat-induced apoptosis (Figure 3E).

Finally, engagement of CD40 promotes recruitment of adapter proteins known as TNF receptor-associated factors (TRAFs) to the receptor cytoplasmic domains [28, 29]. TRAF signaling relies on a series of ubiquitylation events which could potentially be modulated by NAE inhibition. CD40 directly associates with TRAF2, 3 and 6, where TRAF2 activates the cIAP to ubiquitylate TRAF3, promoting its degradation. While CD40L stimulation induced expression of NFκB-regulated cIAPs [30, 31] in CLL cells, NAE inhibition did not alter their expression, suggesting that CD40/TRAF signaling is unperturbed when NAE is inhibited (Figure 3D).

Thus, pevonedistat sensitized primary CLL cells to exogenous death receptor ligands.

### Pevonedistat enhances caspase-8 activation in neoplastic B-cells

**Figure 4:**
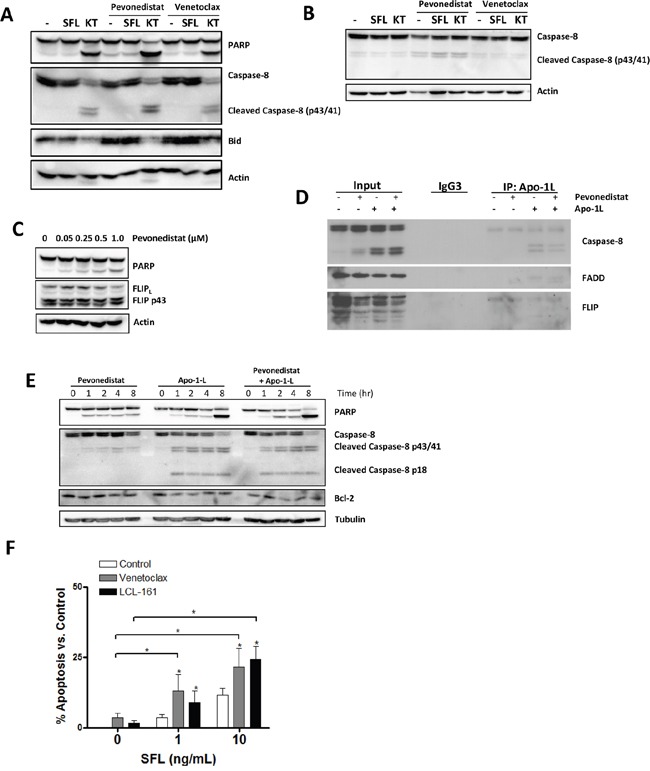
NAE inhibition enhances caspase-8 activation in neoplastic B-cells **A.** OCI-LY3 cells were incubated with 0.5 μM pevonedistat, venetoclax or vehicle control for 24 hours, followed by treatment with 10 ng/mL KT or 10 ng/mL SFL for 1 hour. Cells were lysed and subjected to immunoblotting. **B.** CLL cells were co-cultured with CD40L-expressing stroma for 24 hours, followed by incubation with 0.5 μM pevonedistat, venetoclax or vehicle control for 24 hours, and then stimulated with 10 ng/mL KT or SFL for 1 hour, lysed and subjected to immunoblotting. **C.** OCI-LY3 cells were incubated with the indicated concentrations of pevonedistat for 24 hours, lysed and subjected to immunoblotting. Representative blots of at least 3 independent experiments are shown. **D.** OCI-LY3 cells were incubated with 0.5 μM pevonedistat or vehicle control for 24 hours, and then stimulated or not with 100 ng/mL Apo-1L. Cells were lysed and subjected to immunoprecipitation using Apo-1L antibody as described in the methods. **E.** OCI-LY3 cells were treated with 0.5 μM pevonedistat, 100 ng/mL Apo-1L, or drug combination. Cells were lysed at the indicated timepoints and subjected to immunoblotting. (E) OCI-LY3 cells were incubated with 1 μM venetoclax, 1 μM LCL-161, and the indicated concentrations of SFL for 24 hours. Apoptosis was assessed by Annexin V staining and values normalized to untreated control. * - p<0.05 compared to untreated control.

We next sought to ascertain if targeting NAE facilitated extrinsic apoptosis in neoplastic B-cells. First, we determined whether pevonedistat promoted caspase-8 activation, a key effector caspase involved in death receptor signaling. OCI-LY3 cells which underwent short-term stimulation with TRAIL (but not Fas ligand) demonstrated rapid caspase-8 cleavage, loss of unprocessed Bid and apoptosis (as evidenced by enhanced PARP cleavage; Figure 4A). Conditioning of either OCI-LY3 or primary CLL cells with pevonedistat, but not Bcl-2 inhibitor venetoclax, enhanced TRAIL-mediated caspase-8 activation (Figure 4A-4B).

TNFR-mediated apoptosis requires assembly of a functional death-inducing signaling complex (DISC), which is composed of several signaling adaptors (e.g., FADD) and leads to caspase-8 and Bid processing. c-FLIP, a protease-deficient caspase homolog, is concurrently recruited to the DISC where it inhibits caspase-8 processing. Thus, the balance between c-FLIP and caspase-8 levels determines the outcome of death receptor signaling [32–34]. c-FLIP is a bona fide NFκB transcriptional target, and targeting NAE reduces c-FLIP mRNA and protein in CD40-stimulated CLL cells (Figure 3D and [15]). By contrast, c-FLIP protein levels were modestly reduced in pevonedistat-treated OCI-LY3 cells (Figure 4C).

We supposed that NAE inhibition could lead to decreased participation of c-FLIP in the DISC, thereby promoting caspase-8 processing. Immune-precipitation experiments employing Apo-1L antibody (FasL) demonstrated DISC assembly and caspase-8 activation, as manifested by generation of its active forms (p41/43) [35], in OCI-LY3 cells following Fas engagement (Figure 4D). Still, pevonedistat did not modulate recruitment of c-FLIP or caspase-8 to the DISC, at least in response to FasL stimulation (Figure 4D). We could not analyze DISC in response to TRAIL because of the technical difficulties of DISC isolation using TRAIL antibody.

Our group and others have shown that NAE inhibition induces apoptosis of the neoplastic B-cells through the rebalancing of the Bcl-2 family members towards the pro-apoptotic BH3-only proteins Bim and Noxa; Cdt1 accumulation, DNA damage and checkpoint activation [15, 17, 36]. Here we observed caspase-8 processing in OCI-LY3 cells after 1 hour incubation with pevonedistat, albeit to a lesser extent than in response to death ligands (Figure 4E). This suggested that pevonedistat could promote casepase-8 cleavage by enhancing intrinsic (mitochondrial) apoptosis. Consistent with that, Bcl-2 inhibitor venetoclax and SMAC-mimetic LCL161 also sensitized OCI-LY3 cells to FasL (Figure 4F).

Thus, pevonedistat augmented extrinsic apoptosis in neoplastic B-cells via enhanced caspase-8 activation.

## DISCUSSION

Survival of the neoplastic B-cells depends on the misbalance between the pro- and anti-apoptotic molecules [37, 38]. Venetoclax, a novel BH3-mimetic which targets the pro-survival protein Bcl-2, has been recently approved for therapy of patients with relapsed/refractory CLL with del (17p), where it is highly efficacious [39]. By contrast, responses to venetoclax are short-lived in NHL, including DLBCL [40]. Thus, novel strategies to re-activate apoptosis are necessary. At this time, multiple clinical trials explore venetoclax in combination with inhibitors of B-cell receptor-associated kinases (e.g., ibrutinib) and CD20-targeting agents (e.g., rituximab) in NHL [41]. Here, extending our earlier findings on feasibility of targeting NFκB in neoplastic B-cells, we propose a potential therapeutic approach which employs NAE inhibitor pevonedistat to facilitate extrinsic apoptosis in lymphoma.

We demonstrate that despite high surface expression of death receptors TRAIL-R1/2 and Fas, ABC-and GC-DLBCL cells exhibit resistance to death ligands. Given the involvement of the NFκB pathway in regulation of death receptor signaling and its anti-apoptotic effect in this setting [7–9], we investigated strategies to promote extrinsic apoptosis through targeting NFκB. Pevonedistat, an investigational NAE inhibitor, abrogates NFκB activity in DLBCL [17]. Indeed, pevonedistat sensitized DLBCL cell lines to death ligands. While constitutive activation of the NFκB pathway has been detected across the DLBCL subtypes, it is indispensable for proliferation and survival of ABC-like DLBCL [12, 42]. Targeting NAE potently inhibits NFκB in ABC-DLBCL; by contrast, GC-like undergo DNA re-replication and damage and checkpoint activation when NAE is inhibited [17]. Consistent with this, ABC-DLBCL showed enhanced apoptosis in response to combined treatment with pevonedistat and death ligands, compared with GC-DLBCL.

Unstimulated primary CLL cells sourced from peripheral blood are resistant to extrinsic apoptosis [34, 43]. In agreement with earlier findings, we demonstrate that CD40L stimulation, which partially mimics the lymph node microenvironment, induces death receptor expression [44]. This renders an opportunity to co-opt extrinsic apoptotic pathways in the CLL microenvironment niche *in vivo*. However, like DLBCL cells, primary CLL cells co-cultured with CD40L-expressing stroma *in vitro* were resistant to death ligands. Our data appears to be in agreement with results by others where withdrawal of soluble CD40L was necessary to achieve complete activation of FasL/Fas system in CLL [44]. Importantly, CD40-stimulated CLL cells were sensitized to death receptor signaling by pevonedistat, which we have shown to be a potent NFκB inhibitor in those cells [15, 16]. Interestingly, neutralization of endogenous death ligands, presumably present in CLL-stromal cell co-cultures, did not protect CLL cells from NAE inhibition, suggesting that the pro-apoptotic activity of pevonedistat did not depend on receptor apoptotic pathways.

It is notable that compared to ABC-DLBCL, CLL cells are less efficiently sensitized to extrinsic apoptosis by pevonedistat. Indeed, several mechanisms which prevent caspase-8 activation and thereby contribute to the ‘dysfunctional’ death receptor signaling have been identified in CLL. Resistance of peripheral blood CLL cells to extrinsic apoptosis may be explained in part by low death receptor expression, accompanied by suboptimal DISC formation in response to TRAIL [34]. High c-FLIP_L_ to caspase-8 ratio, accompanied by inefficient processing of the latter, may further contribute to resistance [34]. Lyn, a Src family kinase constitutively activated and involved in B-cell receptor signal transmission in CLL cells, has also been implicated in resistance to extrinsic apoptosis: Lyn induces phosphorylation of procaspase-8, thereby promoting the formation of enzymatically inactive homodimer [45]. It is possible that some of the above mechanisms may underlie DLBCL cell resistance to death receptor agonists as single agents.

Our data suggests that NFκB contributes to resistance to death receptor signaling in neoplastic B-cells, which can be partially reversed by NAE inhibition. c-FLIP, a bona fide NFκB transcriptional target, participates in DISC assembly and counterbalances caspase-8 activation [32, 33]. Interestingly, NAE inhibition had differential effects on c-FLIP expression in DLBCL cells and primary CLL cells, where its downregulation was modest in the former and pronounced in the latter. Treatment with pevonedistat did not seem to enhance DISC formation or c-FLIP participation in the DISC in response to FasL in OCI-LY3 cells. However, it is important to note that NAE inhibition enhanced caspase-8 processing in response to TRAIL more efficiently, and thus enhanced DISC formation cannot be fully excluded.

CD40L stimulation of the neoplastic B-cells derived from the lymph nodes of patients with follicular lymphoma induced NFκB, accompanied by rapid upregulation of Bcl-xL and c-FLIP, leading to TRAIL resistance [46]. CD40L stimulation induces anti-apoptotic Bcl-2 family members in primary CLL cells, including Bcl-X, Bcl-2 and Mcl-1 [15, 47, 48]. NAE inhibition leads to rebalancing of the Bcl-2 family proteins in favor of apoptosis, accompanied by a downregulation of the pro-survival Bcl-2 family members and an increase in pro-apoptotic BH3-only proteins Bim and Noxa [15, 17]. We found that treatment with pevonedistat alone was able to initiate caspase-8 cleavage. Thus, it is possible that NAE inhibition enhances caspase-8 processing through a feedback loop mediated by activation of caspase-3 via intrinsic (mitochondrial) pathways [49].

Other strategies to enhance sensitivity of the neoplastic B-cells to extrinsic apoptosis have been reported. Histone deacetylase inhibitors enhance FADD recruitment to TRAIL-R1 and thereby sensitize CLL cells to TRAIL-apoptosis [50]. The proteolytic activity of caspase-3 can be inhibited by X-linked inhibitor of apoptosis (XIAP) and a novel class of drugs called second mitochondria-derived activator of caspase (SMAC)-mimetics directly bind and antagonize XIAP, sensitizing CLL cells to TRAIL [51, 52]. Indeed, we found that both Bcl-2 inhibitor venetoclax, and SMAC-mimetic LCL-161 sensitized OCI-LY3 cells to FasL, suggesting that those represent viable potential combination strategies in lymphoma.

Pevonedistat has previously been shown to synergize with TNFα to induce apoptosis in monocytes, dendritic cells and hepatocytes via induction of both apoptotic and necroptotic pathways [53, 54]. This may raise concerns regarding synergistic toxicity. However, in a recently reported Phase I study of pevonedistat in NHL, grade≥3 transaminase increases were observed in less than 5% of patients (2/44) [19]. Nevertheless, we would advocate for a careful dose escalation schema and a period of observation following administration of TRAIL agonists in combination with pevonedistat in a clinical trial setting.

Additional investigations are needed to better define how to best use pevonedistat in patients with B-cell malignancies. We have previously reported that pevonedistat enhances the pro-apoptotic effect of the DNA-damaging agent bendamustine in CLL cells [36]. However, in the current era, ‘targeted’ therapies are receiving widespread use in lymphoid malignancies, and are notable for their favorable toxicity profile compared with standard chemotherapy drugs [55]. Thus, combination strategies which involve inhibitors of B-cell receptor-associated kinases and pevonedistat, as previously reported by us [15], seem most attractive.

In summary, our data in primary neoplastic B-cells is consistent with findings by others that pevonedistat enhances the anti-tumor activity of TRAIL in solid tumor cell lines [56], thus suggesting that pevonedistat should be further investigated in therapy of CLL and NHL.

## MATERIALS AND METHODS

### Primary CLL samples and stromal cell co-cultures

Peripheral blood was obtained from patients with CLL at Dartmouth-Hitchcock Medical Center and Oregon Health and Science University following approval by the respective Institutional Review Board and written informed consent of patients. Of 25 patients who provided samples for this study, 80% of patients were previously untreated. 40% had del(13q), 20% - trisomy 12, 24% had no chromosomal abnormalities as detected by FISH; this information was not available in the remaining patients. Peripheral blood mononuclear cells (PMBCs) were isolated using standard Ficoll-Hypaque techniques (Amersham, Piscataway, NJ). Such CLL samples contained ≥90% CD5^+^/CD19^+^ cells, as determined by flow cytometry. CLL cells were cultured in RMPI-1640 supplemented with 15% fetal bovine serum, 100 U/mL penicillin, 100 μg/mL streptomycin, 2 mM L-glutamine, 25 mM HEPES, 100 μM non-essential amino acids and 1 mM sodium pyruvate (Lonza, Walkersville, MD). All experiments were performed with freshly isolated CLL cells.

The mouse fibroblast cell line (L cells) engineered to express CD40L (L4.5) was given to us by Dr. Sonia Neron (Université Laval, Quebec, Canada)[57]. Parental L cells were obtained from American Type Culture Collection (Manassas, VA). Both cell lines were maintained in DMEM with 10% FBS and penicillin-streptomycin. CLL cells were cultured under standardized conditions on stromal cells as previously described [15]. Briefly, stromal cells were seeded to achieve 80-100% confluence; on the following day, CLL cells were plated at a 50:1 ratio and incubated at 37°C in 5%CO_2_. At harvest, CLL cells were gently washed off the stromal layer. When collected for protein analysis, CLL cells were transferred to a new plate and incubated for an additional 60 minutes to allow re-attachment of stromal cells and thus minimize contamination of CLL cells.

DLBCL cell lines Nu-DUL-1, SU-DHL4, SU-DHL6, SU-DHL10 and SU-DHL16 were obtained from American Type Culture Collection (Manassas, VA); OCI-LY18, U-2932 and VAL - from DSMZ (Braunschweig, Germany); OCI-LY3 and OCI-LY19 cells were a kind gift from Dr. Andrew Evens (Tufts University).

### Cell viability testing and drugs

Cell apoptosis was measured in duplicate as previously described using the ApoScreen Annexin V Apoptosis Kit [58]. Briefly, cells were resuspended in 150 μL of Annexin V binding buffer containing 1 μL each of Annexin V-PE, 7-aminoactinomycin (7-AAD) and CD19-FITC (Southern Biotech, Birmingham, AL), followed by flow cytometry on a MACSQuant (Miltenyi Biotec, San Diego, CA).

To measure cell proliferation, cells were plated in 96-well plates (3000/well in 100 μL, 6 wells per sample) with drugs and incubated for 48 hours at 37°C in 5% CO_2_. After incubation, relative numbers of viable cells were measured using a tetrazolium-based colorimetric assay (CellTiter AQueous One Solution Cell Proliferation Assay; Promega, Madison, WI).

The following drugs and antibodies were used: pevonedistat (provided by Millennium Pharmaceuticals Inc., Cambridge, MA); venetoclax (ABT-199, Activ Biochem, Maplewood, NJ), both diluted in DMSO; Fas agonists Apo-1L and SuperFasLigand (SFL; Enzo Life Sciences, Framingdale, NY); KillerTRAIL (KT; Enzo Life Sciences), diluted in sterile water; pan-caspase inhibitor QVD-OPh (Sigma Aldrich, St. Louis, MO, diluted in DMSO); neutralizing antibodies against FasL (CD178), TRAIL (CD253) and TNF-α (BD Biosciences, San Diego, CA). Fluorochrome-conjugated antibodies to CD95 (Fas), CD261 (DR4, TRAIL-R1), CD262 (DR5, TRAIL-R2), CD263 (TRAIL-R3) and CD264 (TRAIL-R4) were purchased from Miltenyi Biotec.

### Immunoblotting and immunoprecipitation (IP)

Proteins were analyzed by immunoblotting as previously described [59]. Briefly, cells were lysed in RIPA buffer (20 mM Tris, 150 mM NaCl, 1% NP-40, 1 mM NaF, 1 mM Sodium phosphate, 1 mM NaVO3, 1 mM EDTA, 1 mM EGTA, supplemented with protease inhibitor cocktail (Roche, Indianapolis, IN), phosphatase inhibitor cocktail 2 (Sigma-Aldrich) and 1 mM phenylmethanesulfonyl fluoride (PMSF). The following antibodies were used: Bcl-2, Bid, cFLIP, cellular IAP (cIAP)-1, cIAP2, caspase-8, FADD, Fas, PARP and cleaved PARP, TRAF2, TRAF3, TRAIL-R2/DR5 (Cell Signaling, Danvers, MA); TRAIL-R1/DR4 (Santa Cruz Biotechnology, Santa Cruz, CA), β-actin, γ-tubulin (Sigma-Aldrich), horseradish peroxidase-conjugated anti-mouse and anti-rabbit antibodies (Cell Signaling).

For DISC IP, OCI-LY3 cells were stimulated with Apo-1L for 1 hour and lysed in immunoprecipitation lysis buffer (150 mM NaCl, 30 mM Tris, 10% Glycerol, 1% Triton-X-100) supplemented as above. Protein lysates were incubated with Apo-1L or isotype control antibody at 4°C overnight, and then with protein A agarose beads (Cell Signaling) at 4°C for 2 hours. Samples were reconstituted in loading dye, heated at 95°C for 5 min and analyzed by immunoblotting, alongside input controls (at 1:10).

### Statistical analysis

Results of individual experiments were analyzed using paired and unpaired Student's *t*-test, Fisher Exact test, non-parametric Mann-Whitney test and Spearman *r*. Statistical analyses were completed using the GraphPad Prism 6 software package (La Jolla, CA). All tests were two-sided, and data were considered to be statistically significant at p<0.05.

## SUPPLEMENTARY MATERIALS FIGURES


