# Supplementary material for: Pevonedistat, a Nedd8-activating enzyme inhibitor, sensitizes neoplastic B-cells to death receptor-mediated apoptosis
Source: Oncotarget. 2017 Feb 3;8(13):21128–39. doi: 10.18632/oncotarget.15050 (PMC5400571; doi:10.18632/oncotarget.15050)
Supplement: Supplementary file 1 [file oncotarget-08-21128-s001.pdf]

Pevonedistat, a Nedd8-activating enzyme inhibitor, sensitizes neoplastic B-cells to death receptor-mediated apoptosis

SUPPLEMENTARY FIGURES

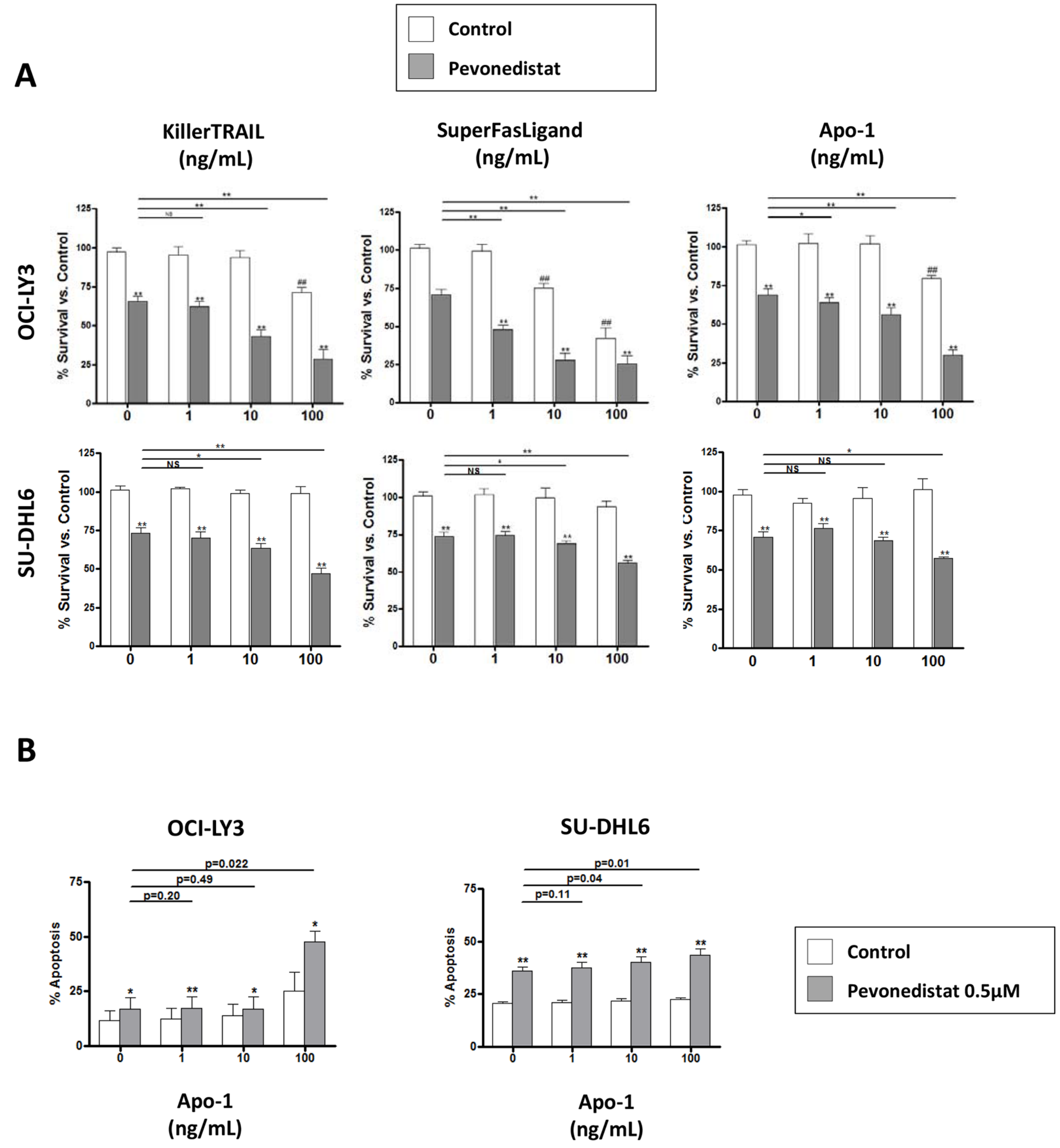

Supplementary Figure 1: Pevonedistat sensitizes DLBCL cells to death receptor agonists. A. Cells were incubated with drugs as shown for 48 hours and subjected to the tetrazolium-based colorimetric assay. Data are the mean ± SE of three independent experiments. ## $p<0.01$ and # - $p<0.05$ compared to untreated control; * - $p<0.05$ and **- $p<0.01$ when comparing death receptor agonist/pevonedistat combination with the agonist alone. **B.** Cells were incubated with the indicated concentration of Apo-1L and 0.5 μM pevonedistat (or vehicle control) for 24 hours. Apoptosis was determined by Annexin V staining. Data are the mean ± SE of three independent experiments. # - $p<0.05$ compared to untreated control; * - $p<0.05$ and ** - $p<0.01$ when comparing Apo-1L/pevonedistat combination with Apo-1L alone.

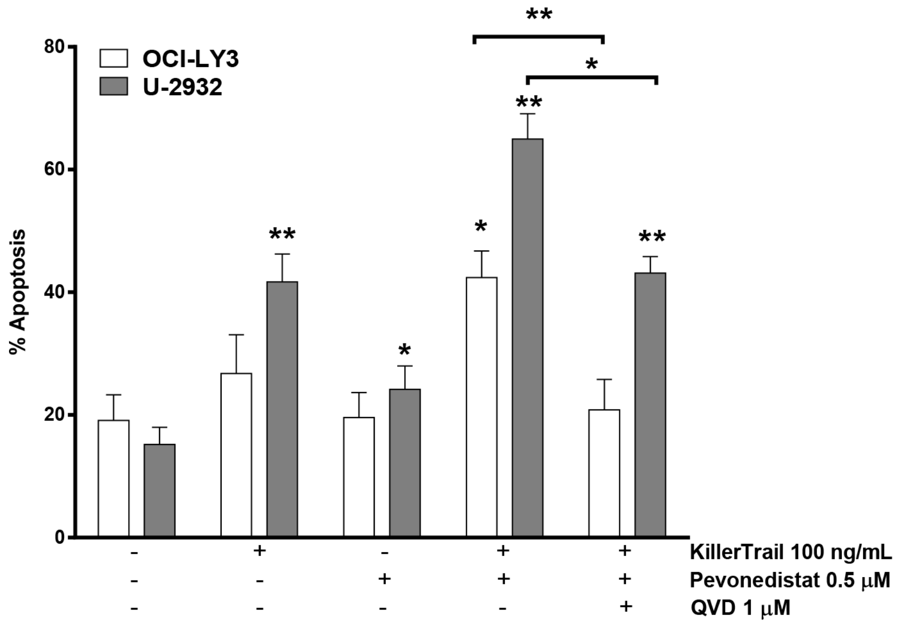

Supplementary Figure 2: OCI-LY3 and U-2932 cells were incubated with 1 μN QVD-OPH for 1 hour and subsequently treated with 100 ng/mL KillerTrail, 0.5 μM pevonedistat or with vehicle control for 24 hours. Apoptosis was determined by Annexin V staining. Data are the mean ± SE of three independent experiments. * - p<0.05 and ** - p<0.01 compared to untreated control.
